# Leukocytosis in Pneumonia

**Published:** 1903-03

**Authors:** 


### Leukocytosis in Pneumonia.

Lambert and Dale (*St. Paul Med. Jour.*, December, 1902,) say as a means of differential diagnosis, the fact that leukocytosis is the normal condition of the blood in pneumonia is often of great assistance. The leukocytosis will help to rule out malaria, typhoid and typhus fever, and acute tuberculous pneumonia, uncomplicated with influenza and the catarrhal pneumonia of influenza.

